# Supplementary material for: Coupled carbon and nitrogen cycling regulates the cnidarian–algal symbiosis
Source: Nat Commun. 2023 Nov 1;14:6948. doi: 10.1038/s41467-023-42579-7 (PMC10620199; doi:10.1038/s41467-023-42579-7)
Supplement: Supplementary file 1 — Supplementary Information [file 41467_2023_42579_MOESM1_ESM.pdf]

Supplementary Information

Coupled carbon and nitrogen cycling regulates the cnidarian-algal symbiosis

Nils Rädecker, Stéphane Escrig, Jorge E. Spangenberg, Christian R. Voolstra, Anders Meibom

Supplementary figures

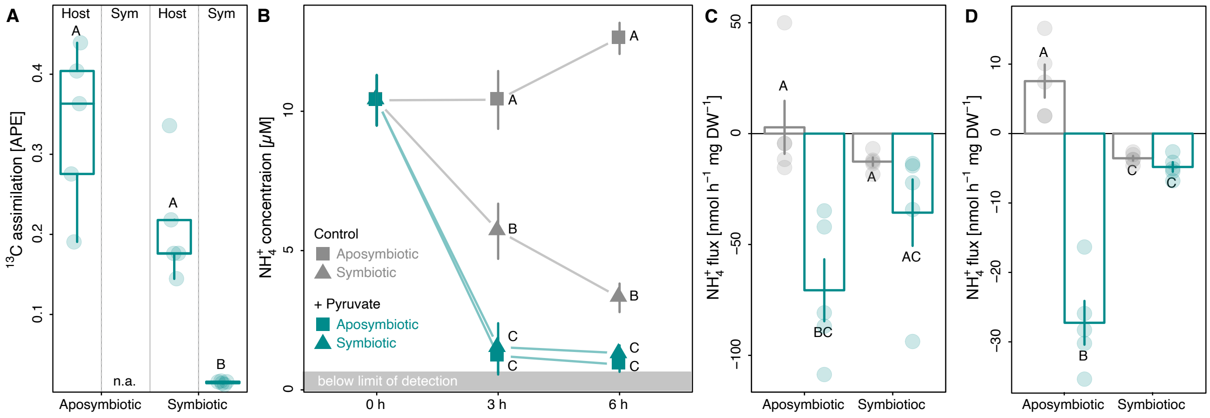

Fig. S1 | Assimilation of pyruvate and its consequences on net ammonium (NH_4^+) uptake in Aiptasia. (A) assimilation of [2,3-^{13}C]-pyruvate in aposymbiotic or symbiotic Aiptasia during 6 h incubations. **(B)** NH_4^+ concentrations during 6 h incubations of Aiptasia. **(C)** Change in NH_4^+ concentrations normalized to Aiptasia dry weight during 3 h of incubation. **(C)** Change in NH_4^+ concentrations normalized to Aiptasia dry weight during 6 h of incubation. Boxplots indicate median, upper and lower quartiles, whiskers show 1.5 x interquartile range. Points/bars with error bars indicate mean ± SEM. Responses for treatments with different letters are significantly different based on Tukey's honest significance test ($p < 0.05$). For each symbiotic state, treatment, and time point 5 Aiptasia were analyzed. APE = atom % excess relative to unlabeled controls, Host = host fraction; Sym = algal symbiont fraction. Source data are provided as a Source Data file.

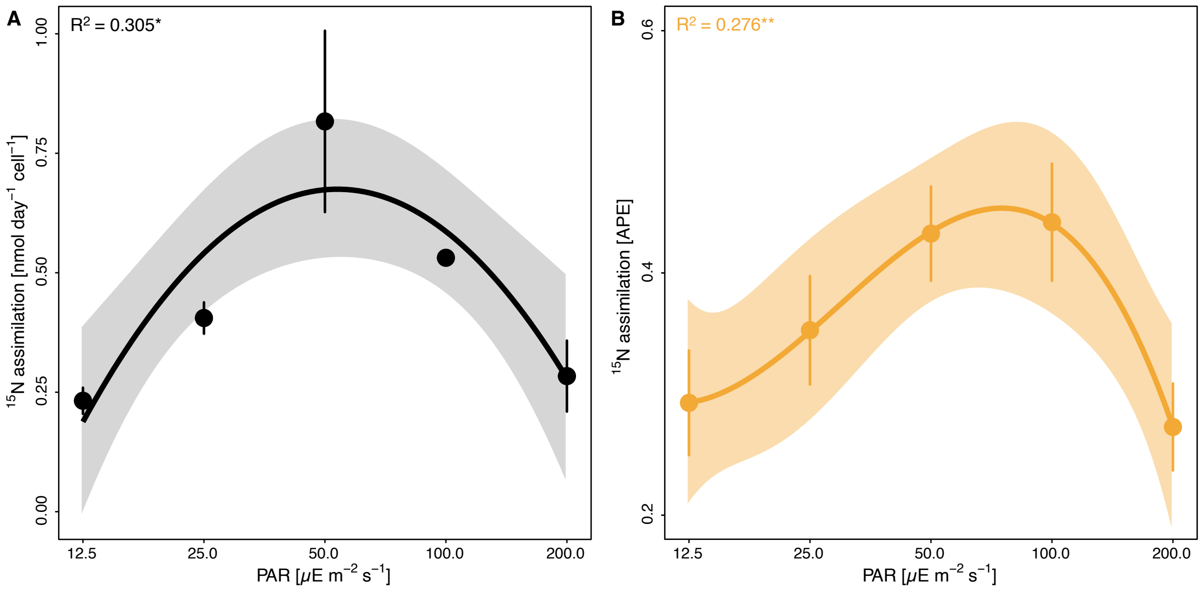

Fig. S2 | Effects of light availability on $^{15}NH_4^+$ assimilation in Aiptasia. (A) Bulk isotope analysis of ^{15}N assimilation in Aiptasia holobionts normalized per algal symbiont. **(B)** NanoSIMS analysis of ^{15}N assimilation in the epidermis of Aiptasia. Points and error bars indicate mean ± SEM; lines represent the best-fitting linear regression models with corresponding 95 % confidence intervals. R^2 values show the proportion of variance explained by the respective model, and asterisks indicate a significant effect of light availability on the response parameter (*p < 0.050, ** p < 0.010). For bulk isotope analysis, 5 Aiptasia were analyzed per light condition. For NanoSIMS analysis, 8 epidermis regions of interest from 2 Aiptasia were analyzed per light condition. APE = atom % excess relative to unlabeled controls, PAR = photosynthetically active radiation. Source data are provided as a Source Data file.

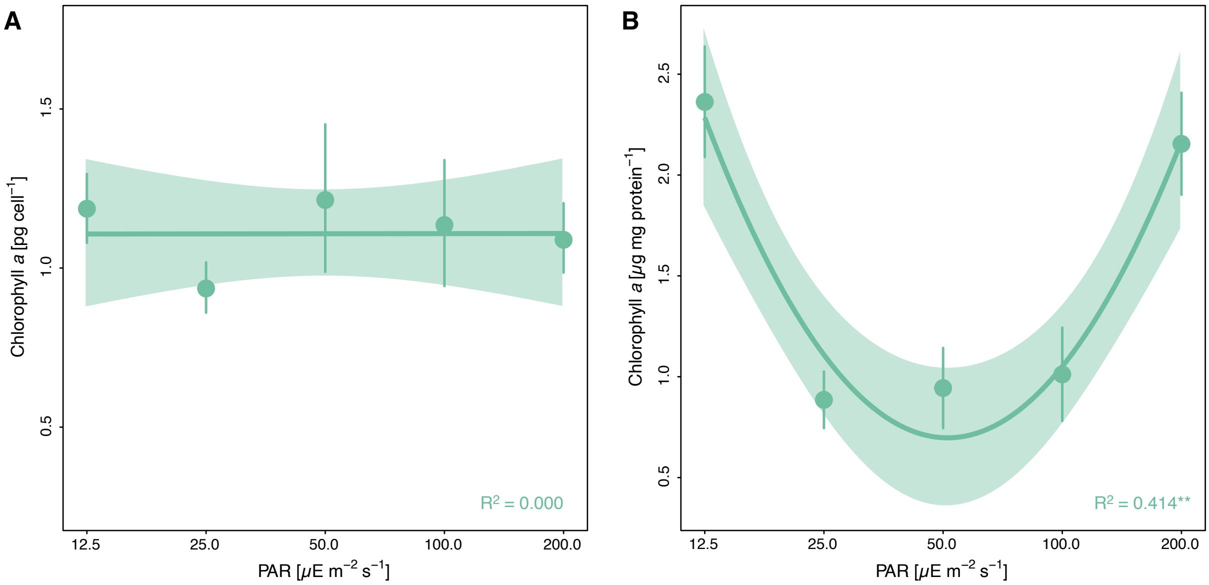

Fig. S3 | Effects of light availability on chlorophyll *a* content of algal symbionts in Aiptasia. Chlorophyll *a* content normalized **(A)** per algal symbiont cell of **(B)** per host protein content. Points and error bars indicate mean ± SEM; lines represent the best-fitting linear regression models with corresponding 95 % confidence intervals. R^2 values show the proportion of variance explained by the respective model, and asterisks indicate a significant effect of light availability on the response parameter (*** p < 0.010). For each light condition, 5 Aiptasia were analyzed. PAR = photosynthetically active radiation. Source data are provided as a Source Data file.

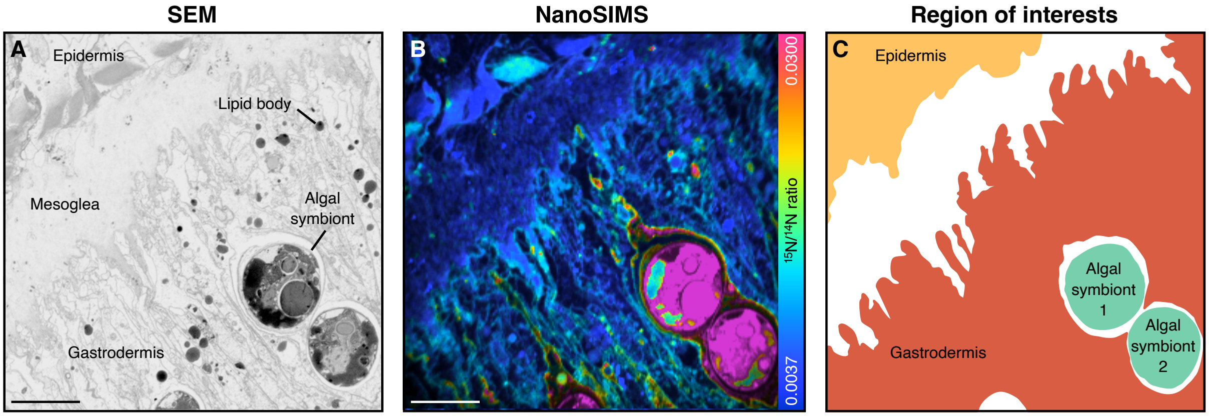

Fig. S4 | Exemplary illustration of NanoSIMS analyses. (A) Scanning electron microscopy (SEM) image of a tentacle cross-section of Aiptasia showing host epidermis, mesoglea, gastrodermis, and algal symbionts. **(B)** NanoSIMS hue saturation image illustrating $^{15}N^{12}C^{-}/^{14}N^{12}C^{-}$ ratios across the same tissue region. **(C)** Corresponding regions of interest selected for the data analysis of host epidermis and gastrodermis as well as individual algal symbionts. Scale bars are 10 µm. For methodological details regarding SEM imaging, please refer to Meibom *et al.* (1).

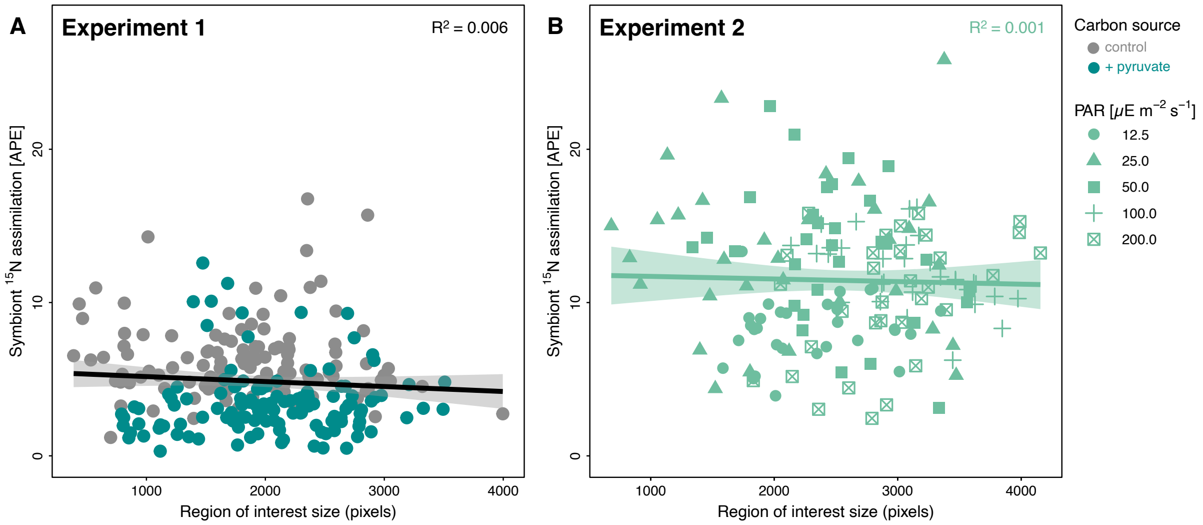

Fig. S5 | Effects of region of interest size on algal symbiont ^{15}N enrichment. The size of algal symbionts (reflected in their number of pixels) in a respective NanoSIMS image had no significant effect on algal ^{15}N enrichment in **(A)** experiment 1 and **(B)** experiment 2. Lines represent the best-fitting linear regression models with corresponding 95 % confidence intervals. R^2 values show the proportion of variance explained by the model. 240 and 160 regions of interest were analyzed for experiments 1 and 2, respectively. APE = atom % excess relative to unlabeled controls. Source data are provided as a Source Data file.

Supplementary references

1. A. Meibom, *et al.,* Correlated cryo-SEM and CryoNanoSIMS imaging of biological tissue. *BMC Biol* **21**, 126 (2023). https://doi.org/10.1186/s12915-023-01623-0
